# Ovarian transcriptional response to *Wolbachia* infection in *D. melanogaster* in the context of between-genotype variation in gene expression

**DOI:** 10.1093/g3journal/jkad047

**Published:** 2023-03-01

**Authors:** Sophia I Frantz, Clayton M Small, William A Cresko, Nadia D Singh

**Affiliations:** Institute of Ecology and Evolution, University of Oregon, Eugene, OR, 97403USA; Institute of Ecology and Evolution, University of Oregon, Eugene, OR, 97403USA; Presidential Initiative in Data Science, University of Oregon, Eugene, OR, 97403USA; Institute of Ecology and Evolution, University of Oregon, Eugene, OR, 97403USA; Presidential Initiative in Data Science, University of Oregon, Eugene, OR, 97403USA; Institute of Ecology and Evolution, University of Oregon, Eugene, OR, 97403USA

**Keywords:** W*olbachia*, transcriptomics, host–microbe interactions, ovary, *Drosophila melanogaster*, *Wolbachia pipientis*

## Abstract

*Wolbachia* is a maternally transmitted endosymbiotic bacteria that infects a wide variety of arthropod and nematode hosts. The effects of *Wolbachia* on host biology are far-reaching and include changes in host gene expression. However, previous work on the host transcriptional response has generally been investigated in the context of a single host genotype. Thus, the relative effect of *Wolbachia* infection versus vs. host genotype on gene expression is unknown. Here, we explicitly test the relative roles of *Wolbachia* infection and host genotype on host gene expression by comparing the ovarian transcriptomes of 4 strains of *Drosophila melanogaster* (*D. melanogaster*) infected and uninfected with *Wolbachia*. Our data suggest that infection explains a small amount of transcriptional variation, particularly in comparison to variation in gene expression among strains. However, infection specifically affects genes related to cell cycle, translation, and metabolism. We also find enrichment of cell division and recombination processes among genes with infection-associated differential expression. Broadly, the transcriptomic changes identified in this study provide novel understanding of the relative magnitude of the effect of *Wolbachia* infection on gene expression in the context of host genetic variation and also point to genes that are consistently differentially expressed in response to infection among multiple genotypes.

## Introduction

*Wolbachia* is a well-studied bacterial endosymbiont that primarily resides in the ovaries of its host and is maternally transmitted. It estimated that 40–60% of arthropod species are infected with a species of the *Wolbachia* genus (Hilgenboecker *et al.* 2008; Zug and Hammerstein 2012). The proportion of infected individuals varies by population, with some populations exhibiting very high frequencies of infection (Weeks *et al.* 2007; Kriesner *et al.* 2013; Kaur *et al.* 2021).

*Wolbachia* exerts a wide range of effects on their various hosts, employing a range of parasitic and mutualistic traits, sometimes even from the same strain. In some host species, *Wolbachia* infection is classified as harmful, causing reduced reproductive output through 4 main mechanisms: cytoplasmic incompatibility (CI), male-killing, feminization, and parthenogenesis (for review see Werren *et al.* 2008). These mechanisms often ensure *Wolbachia*'s propagation to the next generation.

Theoretical and empirical evidence also suggests these parasitic interactions can evolve to become more mutualistic over time (Ballard 2004; Weeks *et al.* 2007). In other cases, *Wolbachia* can confer advantages such as increased reproductive output and protection from viral infection (Vavre *et al.* 1999). Some hosts receive essential nutrients from *Wolbachia* (Brownlie *et al.* 2009; Hosokawa *et al.* 2010), and *Drosophila paulistorum* (*D*. *paulistorum*) even presents an extreme example of obligate mutualism in which *Wolbachia* is needed for gonad development (Miller *et al.* 2010).

The effect of *Wolbachia* therefore strongly depends on the particular host species and genotype as well as the strain of *Wolbachia*. For example, in its *D. melanogaster* host, *w*Mel induces only weak CI in laboratory strains and even weaker CI in the wild (Hoffmann *et al.* 1998). Therefore, the high global infection rate of *w*Mel (estimated 34% in wild populations of *D. melanogaster*) cannot be explained by reproductive manipulation through CI alone and may instead be explained by benefits conferred to the host (Solignac *et al.* 1994; Serga *et al.* 2014). Benefits such as increased survival and fecundity have been documented in *w*Mel-infected strains of *D*. *melanogaster* (Fry and Rand 2002; Fry *et al.* 2004; Singh 2019).

The effects of *Wolbachia* on host gene expression have been the focus of recent attention. Studies in the ovaries (He *et al.* 2019), whole body (Detcharoen *et al.* 2021; Lindsey *et al.* 2021), and testis (Dou *et al.* 2021) have revealed many genes and pathways that are differentially transcribed in response to *Wolbachia* infection. However, these studies have been conducted in single host genetic backgrounds. As a consequence, it is yet unknown if host transcriptional response to *Wolbachia* infection is genotype dependent and, if so, how the magnitude of the transcriptional response to infection compares to the magnitude of inter-strain variability in host gene expression.

To address these open questions, we performed RNA sequencing and differential expression analysis to compare the transcriptomes of *Wolbachia*-uninfected and infected flies of 4 different host strains. Since the ovaries are the primary site of *Wolbachia* colonization, we sequenced the ovarian transcriptome. We focused primarily on characterizing global ovarian transcriptome changes in response to *Wolbachia* infection. The use of 4 different strains of *D. melanogaster* allowed us to disentangle transcriptional variation due to infection, genotype, and the interaction of infection and genotype. We note that all 4 strains of *D. melanogaster* are infected with *w*Mel haplotype I. Though there are certainly genetic differences among the *w*Mel haplotype I strains, these differences are limited, and the levels of genetic variation within these *Wolbachia* strains are far lower than the genetic variation between fly strains (Richardson *et al.* 2012).

Our experimental design therefore allows us to focus on host genotype, infection status, and interaction effects. Our results suggest the contribution of *Wolbachia* to ovarian transcriptional variation is limited, variable, and mediated by the host genotype. Finally, we identified a subset of genes with functions related to cell division, translation, and metabolism that are differentially regulated under *Wolbachia* infection.

## Methods

### *Drosophila* stocks

We used 4 strains from the Drosophila Genome Reference Panel (DRGP) (Mackay *et al.* 2012) for this study: RAL73, RAL783, RAL306, and RAL853. These lines have standard chromosome arrangements and are naturally infected with *Wolbachia pipientis* (*W. pipientis*) (Huang *et al.* 2015). Genomic analysis confirmed that these flies are infected with *w*Mel haplotype 1 (Richardson *et al.* 2012). To generate *Wolbachia*-uninfected flies, we raised these strains on food containing tetracycline at a concentration of 0.25 mg/mL media for 2 generations. To reintroduce the natural microbiome without *Wolbachia*, *Wolbachia*-uninfected flies were subsequently raised on standard food for at least 10 additional generations before transcriptome data were obtained (Singh 2019). Our standard fly food is the Bloomington Drosophila Stock Center Standard Cornmeal Medium. *Wolbachia* infection status was confirmed prior to experimentation using a PCR-based assay with *Wolbachia*-specific primers targeted to *Wolbachia*-specific protein (Jeyaprakash and Hoy 2000). *Wolbachia* infection status was also confirmed for each sample after RNA sequencing by testing for reads that aligned successfully to the *Wolbachia* reference genome. Throughout their rearing, flies were kept at 25 °C on a 12:12 h light–dark cycle.

### Ovary dissections and sequencing

We placed carbon dioxide-anesthetized 3-day-old virgin females in phosphate-buffer saline, dissected their ovaries, and froze the ovaries on dry ice. We prepared 4 replicates of each of the 4 genotypes for each infection status, with 10 pairs of pooled ovaries comprising each replicate. Dissections, library preparations, and sequencing were performed in 2 batches 2 months apart: all dissections on the RAL783 and RAL73 samples were performed on 1 day, and all dissections of RAL306 and RAL853 were performed in 1 day 2 months later. Each replicate of 10 pairs of ovaries remained frozen until total RNA was isolated from them using the Zymo Direct-zol kit. The integrity of the isolated RNA was evaluated using an Agilent Fragment Analyzer, and RQN values ranged from 8.9 to 10. Poly-A-selected mRNA-seq libraries were prepared using the NuGen (Tecan) Universal Plus kit, which introduces chemical fragmentation of the mRNA prior to first strand synthesis. RNA isolations and RNA-seq libraries were prepared and sequenced at the University of Oregon Genomics and Cell Characterization Core Facility. Libraries were sequenced on a HiSeq 4000 using single end, 150-bp reads.

### Sequence processing and alignment

Raw reads were aligned to the *D*. *melanogaster* reference genome from the Berkeley Drosophila Genome Project (BDGP) assembly release 6 with STAR aligner v. 2.5.3a (Dobin *et al.* 2013) with default parameters. Batch 1 (comprised of strains RAL73 and RAL783) generated an average of 22,443,144 reads with 98% aligning uniquely to the reference genome, and batch 2 (comprised of RAL306 and RAL853) generated on average 21,936,370 reads with 89% aligning uniquely to the reference genome (Supplementary Table 1).

### Differential gene expression analysis

**Fig. 1.**
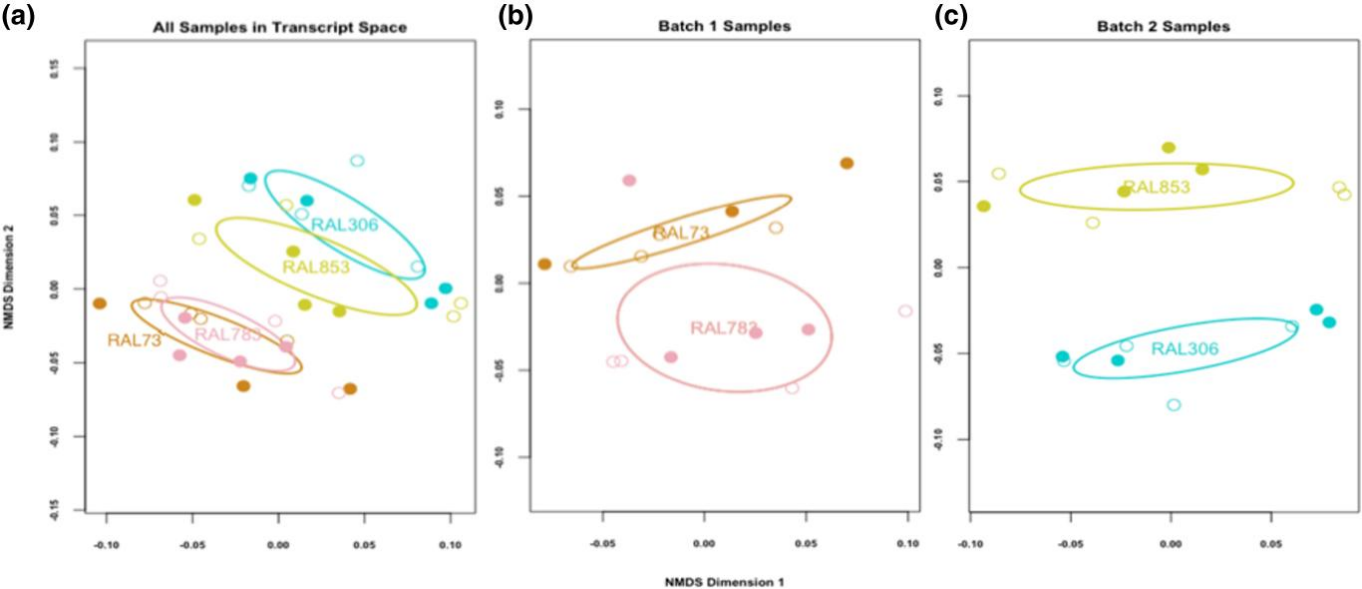
Global ovarian transcriptome nMDS plots. Each sample is represented in 2-dimensional transcript space with a circle, with open circles representing uninfected samples and filled circles representing infected samples. Each genotype is depicted with a different color. The ellipses mark the 95% confidence intervals of the centroid for the samples from each genotype. Note that nMDS dimensions do not quantify distances between points in the space, just their ranks. a) Genotype contributes to the most obvious clustering among samples. The batch effect is also visible: the RAL73 and 783 samples form a cluster while the RAL306 and RAL853 samples form a separate cluster. b) Dissimilarity was calculated only between samples in batch 1. Samples cluster by genotype and subtly by infection status for RAL73. c) Dissimilarity was calculated only between samples in batch 2. The samples group by genotype and there is no obvious grouping by infection status.

All analyses were conducted using R (v. 3.4.3) packages. For each sample, we counted the number of uniquely mapped reads using GenomicAlignments (v. 3.6-intel-2017b) (Lawrence *et al.* 2013) with default parameters. We then used DESeq2 (Love *et al.* 2014) to perform normalization and differential expression analysis on gene count data. Specifically, we fit a model of expression level for each gene as determined by the effect of infection status, while controlling for genotype and the interaction of infection status and genotype. DESeq2 uses a model particularly suited for the overdispersion of RNA-seq data based on the negative binomial distribution. We set the uninfected samples as the base level, so “upregulation” refers to higher normalized read counts in the infected flies. DESeq2 uses the Benjamini and Hochberg (Hochberg and Benjamini 1990) method by default to control the false discovery rate (FDR). We define differentially expressed (DE) genes after controlling the FDR at 0.05. We did not enforce a log_2_ fold-change threshold on top of the FDR criterion based on the premise that genes in the recombination pathway are precisely regulated so that even a small change in expression levels could reflect a functional change in core processes such as meiosis (Reynolds *et al.* 2013; Ziolkowski *et al.* 2017). Using the model above, we conducted gene-wise differential expression tests separately for libraries from batch 1 and batch 2, because genotypes were not replicated across batches. Given this design property, and the clearly strong transcriptome-wide batch effect (Fig. 1), performing the analyses separately for each batch allowed us to properly evaluate effects of genotype (and genotype-by-*Wolbachia* interaction) on gene expression independently of batch.

### Multivariate analysis with nMDS and perMANOVA

To visualize differences in expression among 17,377 transcripts across all samples, we used Bray–Curtis dissimilarity to generate overall and batch-specific non-metric multidimensional scaling (nMDS) ordination plots. We quantified the percentage of dissimilarity explained by infection status, genotype, and the interaction of infection and genotype with a permutational multivariate analysis of variance (perMANOVA) test. Similar to the gene-wise differential expression analyses above, we accounted for batch by performing the perMANOVA test separately on each of the 2 batches and accounted for genotype within each test by limiting permutations to samples within genotypes. We used the R package vegan v. 2.5-5, (Oksanen *et al.* 2007) to calculate distances, plot nMDS, and perform the perMANOVA test.

### Outlier ordination

Initial visualization with an nMDS plot revealed that sample RAL73 *Wolbachia*-infected A (RAL73 w + A) differed from the other 3 replicates of RAL73-infected samples, as well as all other samples in this study. To determine whether this sample represented an outlier with significant influence on the results of the differential expression analysis, we examined Cook's distance on the normalized count data, as calculated by DESeq2. A higher Cook's distance for a sample indicates more leverage in determining a given transcript's fold change. We compared the average Cook's distance for each sample across all transcripts and the number of times each sample contained the highest Cook's distance for each transcript (Supplementary Fig. 1). Replicate samples should not differ much in their distribution of Cook's distances, but we found that the average Cook's distance for sample RAL73w + replicate A was much higher than all other samples and most often contains the maximum Cook's distance across all transcripts. These results suggest sample RAL73w + A has aberrant expression of many genes. Although DESeq2 can manage outliers for transcripts of genes if there are replicates of each factor, we decided that a sample which consistently contains an outlier across many genes likely represents not true biological signal but rather an error of preparation. This distribution of Cook's distances led us to exclude RAL73w + A from all subsequent analyses.

### Gene ontology analysis

We identified Gene Ontology (GO) descriptions for the differentially expressed genes using the Panther biological process classification system, as implemented in Panther's data mapping online tool (Thomas *et al.* 2003). Panther classifies each GO term into nested levels, and we report the level of nesting for GO terms in order to provide more specific information. We then tested whether any GO terms were overrepresented in each group of differentially expressed genes using Panther's statistical overrepresentation test, which uses a Fisher's exact test with FDR controlled at 0.1 and all annotated genes in the *Drosophila melanogaster* genome as reference.

### Distribution of differentially expressed genes across genome

To test whether differentially expressed genes are randomly distributed across the genome, we performed a non-parametric runs test for randomness (Bradley 1968). We categorized each gene as differentially expressed or not, based on the same criteria of FDR controlled at 0.05. We then placed each gene's differential expression information in order of placement across the genome, using the gene start site as the location. A switch from differentially expressed to not, or vice-versa, was considered a complete run. We visualized these patterns of gene expression across the genome with the R package karyoploteR (Gel and Serra 2017) version 1.8.8. For each chromosome, we depict the differentially expressed genes at their location in the BDGP6 *D. melanogaster* reference genome.

## Results

### *Wolbachia*'s influence on ovary transcriptional differences is variable while the influence of genotype is consistent across samples

To understand the relative extent to which transcriptional variation depends on *Wolbachia* infection status and genotype, we visualized this variation across samples using nMDS (Fig. 1a). Arguably the strongest signal in the nMDS space is a batch effect. In our experimental design, we conducted the experiment at 2 different times. In the first experiment, we collected and sequenced flies for strains RAL73 and RAL 783. In the second experiment, we did the same for RAL306 and RAL856. The batch effect in the analysis therefore reflects the time at which the experiment was conducted, and this is reflected in the nMDS space. Specifically, the cluster of samples prepared in the first batch (RAL73 and RAL783) is distinct from the cluster of samples prepared in the second batch (RAL306 and RAL856) and indeed show stronger evidence for separation in nMDS space than any other grouping. Isolating this batch effect allows us to analyze biological patterns within each batch, which can be treated as 2 separate but parallel experiments.

To explore the hypothesis that genotype is also contributing to the separation of the samples in batch 1 from the samples in batch 2, we examined pairwise relatedness among the DGRP strains used in the experiment. As reported previously, nearly all of the DGRP strains are unrelated (Mackay *et al.* 2012). The distribution of pairwise relatedness has a major peak around 0, and only 11 pairs have a genetic relationship of 0.5 or greater. None of our strains show pairwise relatedness anywhere near this level (Supplementary Table 2). In fact, the largest relatedness value is very low (0.047; lines 73 and 783.) We do note that the strains that are closest together on the nMDS plot are lines 73 and 783, which could suggest that there is an effect of genotype on the major separation between samples in batch 1 from the samples in batch 2. However, as noted above, a relatedness of 0.047 is quite small. Moreover, lines 306 and 853 also cluster together, and their genetic relatedness is −0.011 (Supplementary Table 2) which indicates that these lines are less related than average. Although our design precludes a formal statistical test of the relative effects of batch and genotype because they are confounded, the relatedness data support the idea that genotype contributes very little if anything to the separation between the samples in batch 1 and the samples in batch 2.

The strongest biological pattern in this analysis is the grouping of samples based on genotype. Samples of the same genotype are often associated with each other regardless of infection status. Both genotype and infection status contribute to transcriptional differences among samples in batch 1 when plotting samples from each batch on a separate nMDS plot (Fig. 1b). In contrast, we find that genotype is a strong determinant of transcriptional differences, while infection status is not in the batch 2 samples alone (Fig. 1c). Overall, the contribution of *Wolbachia* to global ovarian transcriptional variation is subtle but depends on genotype, while genotype consistently accounts for transcriptional differences in these samples.

To statistically test and quantify the patterns observed in the nMDS plot, we used permutational multivariate analysis of variance (perMANOVA) to estimate the percent of variation in gene expression explained by infection status, genotype, and the interaction of these factors (Table 1). Consistent with the nMDS patterns, genotype accounts for a large and statistically significant proportion of variation in both batch 1 (*R*^2^ = 0.17, *P* = 0.006) and batch 2 samples (*R*^2^ = 0.26, *P* = 0.01). Interestingly, for batch 1, infection status also explains a significant percent of variation (*R*^2^ = 0.19, *P* = 0.003). In batch 1, the interaction of genotype and infection also explains a statistically significant proportion of transcriptional variation (*R*^2^ = 0.13, *P* = 0.02). This interaction means *Wolbachia*'s contribution to transcriptional differences in these samples depends on host genotype (for RAL73 or RAL783), and could be obscuring obvious clustering by infection status in the nMDS plots in Fig. 1. Also, 1 observation in batch 1 (the RAL783 individual with the largest *y*-axis value in Fig. 1b) likely contributes to an apparent inconsistency between nMDS pattern and perMANOVA results because it reduces average dissimilarity between genotypes but increases average dissimilarity between treatments within RAL783.

**Table 1.** Summary of perMANOVA test results

Factor	Batch 1					Batch 2				
	df	meanSS	*F*-value	*R* ^2^	*P*-value	df	meanSS	*F*-value	*R* ^2^	*P*-value
Infection	1	0.007	4.08	0.19	0.003	1	0.003	1.10	0.057	0.3
Genotype	1	0.006	3.66	0.17	0.006	1	0.015	5.02	0.26	0.01
Infection:genotype	1	0.005	2.73	0.13	0.024	1	0.004	1.22	0.063	0.28
Residuals	11	0.002		0.51		12	0.003		0.62	

df—degrees of freedom, meanSS—mean sum of squares. *P*-values are based on 1,000 permutations (lowest possible *P*-value is 0.001).

Infection status and genotype explains a large and significant proportion of the dissimilarity among samples in batch 1 while genotype alone explains the largest proportion of accounted dissimilarity in batch 2. The perMANOVA is based on Bray–Curtis dissimilarities using gene expression differences across samples. The test was performed separately for each batch.

In contrast, infection status accounts for a smaller and non-significant proportion of transcriptional variation among samples in batch 2 (*R*^2^ = 0.06, *P* = 0.3). Consistent with the small effect of infection status, the interaction effect for the samples in batch 2 was also small and non-significant (*R*^2^ = 0.06, *P* = 0.28). In summary, genotype and infection status contribute about equally to the variation in transcription among the samples in batch 1, whereas genotype accounts for much more of the variation than does infection status in batch 2. Therefore, while the contribution of infection status to host gene expression is variable, the contribution of genotype is consistently large and significant in these samples. We note that the residuals explain a large percentage of the observed variation in gene expression. Though some of this unexplained variation is probably technical in nature, many biological factors including host microbiome and *Wolbachia* titer could be contributing to the observed gene expression variation.

### *Wolbachia* infection induces differential gene expression in *D. melanogaster* ovaries

The transcriptional profile of *Wolbachia* infection can also be described by the individual genes that are differentially regulated in *Wolbachia*-infected flies as compared to uninfected flies. In batch 1, 1,104 genes (6% of all transcripts) are differentially expressed, with 618 upregulated in *Wolbachia*-infected flies and 486 downregulated. The differentially expressed genes, along with their fold change, direction of change, mean number of counts, test statistic, and adjusted *P*-values sorted in ascending order are shown in Supplementary Table 1. In batch 2, 96 genes (0.5% of all transcripts) are differentially expressed, with 36 upregulated and 60 downregulated genes (Supplementary Table 2). This difference between the number of genes identified in the 2 batches is consistent with the multivariate analysis, which shows that infection status does not account for a large proportion of global transcriptional differences in batch 2. By comparing the sets of genes from the 2 batches we find only 26 genes that are consistently differentially expressed across these samples despite genotype and batch differences (Supplementary Table 3). This limited overlap may be driven by the small number of differentially expressed genes in batch 2, or by the possible interactions of genotype and infection with respect to between-batch genotype comparisons, which are not testable given our experimental design.

### Differentially expressed genes are randomly distributed across the genome

**Fig. 2.**
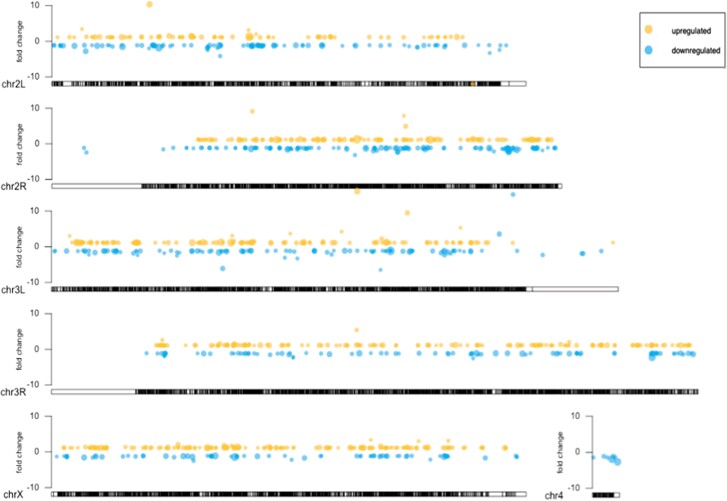
Location of differentially expressed genes from batch 1 across the genome for each chromosome. Differentially expressed genes are distributed randomly across the genome as determined by the runs test for randomness. Genes with an adjusted *P*-value <0.05 are shown at their position on the chromosome, represented by the ideogram. Each circle represents a gene. The placement of the circle on the fold change axis shows the value of fold change difference when comparing uninfected and infected samples. The size of the circle is proportional to the range of *P*-values for all DE genes, with the biggest circles corresponding to the smallest *P*-values.

In the *Drosophila* early ovarian transcriptome, genomic regions with higher rates of transcription also have higher rates of recombination (Adrian and Comeron 2012). Since *Wolbachia* infection leads to increased recombination at certain genomic regions (Mostoufi and Singh 2022) (Singh 2019; Bryant and Newton 2020), we tested whether infection may also be associated with altered levels of transcription at certain genomic regions. To do so, we performed runs test for randomness (Bradley 1968) to determine whether the binary sequence of genes that are categorized as differentially expressed is random across the genome. We performed a runs test for both batches for each chromosome arm and failed to reject the null hypothesis of randomness in all cases, suggesting that the location of differentially expressed genes across the genome is random. This random placement of differentially expressed genes across each chromosome arm is illustrated in Fig. 2 for batch 1 as an example. The visualization and runs test results provide no evidence that *Wolbachia* infection is associated with differential expression of genes at particular genomic locations.

### Differentially expressed genes are enriched in cell division processes

To better understand the function of these differentially expressed genes, we associated each gene with its most nested biological process GO terms (Thomas *et al.* 2003; Mi *et al.* 2010) and tested for overrepresentation of these terms. In batch 1, the 1104 differentially expressed genes contained significant overrepresentation of 95 GO terms and underrepresentation of 4 GO terms. These GO terms are provided in Supplementary Table 5, sorted in order of highest to lowest enrichment score. The 3 most overrepresented terms are “spindle midzone assembly” (enrichment = 12.89, FDR = 0.008), “cytoplasmic translation” (enrichment = 8.56, FDR = 0.0001), and “mitotic spindle elongation (enrichment = 8.06, FDR = 0.027).” Among the enriched terms were other cell cycle-related processes such as “centrosome separation” (enrichment = 6.78, FDR = 0.001) and “meiotic cytokinesis” (enrichment = 3.15, FDR = 0.033). Other common terms were those related to translation, such as “ribosomal small subunit assembly” (enrichment = 6.45, FDR = 0.001).

Since batch 2 has only 96 differentially expressed genes, the statistical power to determine overrepresentation of GO terms was limited. Using an FDR threshold of 0.1, no GO terms were significantly enriched in batch 2. However, to get a sense of the gene processes associated with differential expression in batch 2, we looked at the 55 GO terms with a raw *P*-value below 0.05 (Supplementary Table 6). The top terms in batch 2 are “*N*-acetylneuraminate catabolic process,” “regulation of histone H4 acetylation involved in response to DNA damage stimulus,” and “maintenance of rDNA” (enrichment > 100, *P* = 0.013 for all 3 terms). GO terms related to cell division processes are not among the most enriched in this small set of genes, but relevant terms include “synaptonemal complex assembly” (enrichment = 26.07, *P* = 0.04) and “female meiosis sister chromatid cohesion” (enrichment = 78.22, *P* = 0.019). Again, none of these terms are significantly enriched once correcting for multiple tests.

## Discussion

### Global transcription patterns suggest a consistent effect of genotype and variable influence of *Wolbachia* infection

Experiments from a wide range of model systems align with our finding that genotype significantly contributes to differences in global transcription (Hutter *et al.* 2008; Poelstra *et al.* 2014) and is consistent with evidence that variation in gene expression is heritable (Brem *et al.* 2002; Dixon *et al.* 2007). In fact, these same *D. melanogaster* strains (ours are a subset of the Drosophila Genetic Reference Panel) were used to quantify transcriptional differences due to genotype (Huang *et al.* 2015). Of 18,140 genes measured in the Huang *et al.* (2015) study, 42% showed significant variability in expression levels due to genotype. In our study, 11% of genes are differentially expressed when comparing RAL73 to RAL 783, and 16% of genes are differentially expressed when comparing RAL306 to RAL853 (while accounting for infection status). The larger percentage found in the Huang *et al.* 2015 study may be attributed to their quantification of variation across 205 strains vs. across 2 strains (per batch) in our study.

In contrast to the consistent effect of genotype, we found that the differences in transcription explained by *Wolbachia* infection were variable between the 2 batches. *Wolbachia* infection explained a small and non-significant proportion of variation in batch 2, but it explained approximately as much variation as genotype in batch 1. These differences in the explanatory power of infection could be due to greater transcriptional differences between samples within RAL306 and RAL853 that limit the power to detect variation due to infection status. The results could also reflect true biological differences in the degree to which *Wolbachia* affect ovarian transcription in different genotypes. A combination of these and other factors could be contributing, and future studies will be needed to disentangle the transcriptional variation due to technical vs. biological differences among the 4 genotypes studied here.

Though we cannot parse out the differences due to genotype vs. batch across all 4 genotypes studied, we can compare the effect of genotype within each batch. We found a significant interaction between genotype and infection status for the samples in batch 1, suggesting the effect of *Wolbachia* on transcription depends on the genotype of the host. That a microbe's effect depends on host genotype is increasingly documented in many systems (Ko *et al.* 2009; Small *et al.* 2017; Mateus *et al.* 2019). The symbioses associated with *Wolbachia* vary widely by host, ranging from commensal to parasitic to mutualistic. Our finding shows that the specificity of *Wolbachia*'s relationship to its host also applies to different genotypes within the same species and that this specificity is reflected at the transcriptional level.

### Additional factors may contribute to global transcription patterns

In addition to host genotype and infection status, 2 additional factors that could contribute to transcriptional variation merit discussion. First, there may be variation in *Wolbachia* titer across the 4 host genotypes studied here. If such variation exists, it could contribute to the observed variation in gene expression across infected flies. Variation in *Wolbachia* titer has been previously shown to affect host phenotype including differential protection to viruses and lifespan (Chrostek *et al.* 2013). Second, it is documented that *Wolbachia* infection can alter the *D. melanogaster* microbiome (Simhadri *et al.* 2017), and it is also possible that different *Wolbachia* titers have differential effects on the host microbiome. It is also possible that the antibiotic treatment used to create our *Wolbachia*-free lines, although over 20 generations ago, has led to permanent alterations to the microbiome. Differences in the microbiome have been associated in changes in gene expression for reproductive and neuronal genes in mated (but not unmated) *D. melanogaster* females. It is therefore possible that there are differences between the microbiomes of our infected and uninfected flies and that these differences contribute to the observed variation in gene expression in the current study.

### Global transcription patterns reflect symbiosis between *D. melanogaster* and *W. pipientis*

We found that *Wolbachia* infection did not explain the majority of transcriptional variation among these samples. Although certain microbial infections significantly alter host transcription of many genes (Camilios-Neto *et al.* 2014; Aprianto *et al.* 2018; Rienksma *et al.* 2019), other infections exert a more limited effect (Rawls *et al.* 2004; Camp *et al.* 2014; Small *et al.* 2017). Parasitic and obligately mutualistic microbes often impose sweeping changes to host transcription. For example, a study of host tissue–specific transcriptional response to the pathogenic bacterium *Yersinia pseudotuberculosis* (*Y. pseudotuberculosis*) revealed substantial changes in expression. In that study, 1,336 genes had at least a 4-fold change in expression (Nuss *et al.* 2017), while our study identified only 210 and 154 genes with this extreme fold change in batch 1 and batch 2. Manipulations of obligately mutual microbes have also revealed sweeping changes to the host transcriptome (Bing *et al.* 2017; Mateus *et al.* 2019). While *Wolbachia* is considered an obligate mutualist in some hosts (Miller *et al.* 2010; Taylor *et al.* 2013) and exerts parasitic effects in other hosts (Bourtzis *et al.* 1996), it does not exhibit this extreme relationship with *D. melanogaster*. The limited and variable transcriptional changes we observe in this study are consistent with this form of symbiotic relationship.

These changes are also consistent with recent work examining the effect of *Wolbachia* infection on gene expression. In comparing whole animal transcriptomes, *Wolbachia* infection was associated with differential regulation of 285 genes that were either differentially expressed or showed significant changes in isoform use (Lindsey *et al.* 2021). Similar patterns were found in germline transcriptomic data and proteomic data (Christensen *et al.* 2016; He *et al.* 2019; Dou *et al.* 2021). This number of differentially expressed genes is on the scale of what we observe in the current study. Genes that show altered patterns of gene expression belong to pathways associated with stress response, transcription and translation, recombination, and cell cycle checkpoint (Lindsey *et al.* 2021), which is similar to what we find as well.

### Lack of overlap among differentially expressed genes

Our results merit discussion particularly with regard to 2 previous studies looking at the effects of *Wolbachia* infection on transcription in whole flies (Lindsey *et al.* 2021) and ovaries (He *et al.* 2019). The whole-body transcriptome data from Detcharoen *et al*. (2021) were not included in this comparison because the study was limited to orthologous genes between *D. melanogaster* and *Drosophila nigrosparsa* (*D. nigrosparsa*). Interestingly, of the genes that are identified as differentially expressed across the 3 studies, only 2 emerge as differentially expressed in all 3 studies: FBgn0046776 and FBgn0051619. The former is a pseudogene and the latter encodes *no long nerve cord*. This is not due to any 1 dataset in particular, as only 9 genes overlap between the He *et al.* (2019) and Lindsey *et al.* (2021) study, 35 genes intersect between the current study and the whole animal Lindsey *et al.* (2021) study, and 6 genes intersect between the current study and the ovarian He *et al.* (2019) study.

At first blush, the lack of overlap may seem surprising. However, given the findings in all 3 studies that only a small fraction of the transcriptome is differentially expressed in response to infection, coupled with our observation that host genotype is the major determinant of gene expression variation, we argue that the lack of overlap is reasonable. Different host strains were used across the studies, and we noted a variable infection effect among the genotypes within our single study. Moreover, other environmental factors were sure to vary as well including diet and maternal age. Our findings suggest that moving forward, for the purposes of reproducibility across labs, testing for the effects of a particular treatment on gene expression should involve multiple, preferably overlapping host genotypes, highly controlled environmental factors, and randomized experimental design.

### Gene Oncology terms provide direction for future experiments investigating cell cycle processes

The percent of the transcriptome affected in our study (6% and 0.5%) is similar to a previous study which also did not find large-scale changes in ovarian transcription. It was recently found that 296 genes (2.2%) were differentially expressed when comparing ovaries of *D. melanogaster* infected and uninfected with *Wolbachia* (He *et al.* 2019). Also consistent with this limited effect, a study of the *D. melanogaster* and *Drosophila simulans* (*D. simulans*) ovarian proteome found that 61/549 proteins (11%) were differentially regulated in *D*. *melanogaster* and 49/449 (11%) were differentially regulated in *D*. *simulans* (Christensen *et al.* 2016). Though these 2 studies and ours are the only ones that specifically focus on the ovarian activity of *D. melanogaster* under *Wolbachia* infection, a number of others have characterized the transcriptomic response to *Wolbachia* infection in various species and tissues. These studies confirm the effect of *Wolbachia* depends on the host, as different processes were found to be affected, including reproduction, immunity, and stress response (Brennan *et al.* 2008; Xi *et al.* 2008; Hughes *et al.* 2011; Zheng *et al.* 2011; Chevalier *et al.* 2012; Kremer *et al.* 2012; Pan *et al.* 2012; Rao *et al.* 2012; Dou *et al.* 2021; Lindsey *et al.* 2021).

The differentially expressed genes from batch 1 are enriched in processes related to mitotic and meiotic cell division. Five of the top 6 most enriched significant GO terms are related to late cell division processes including chromosome segregation: “spindle midzone assembly,” “mitotic spindle elongation,” “kinetochore organization,” “attachment of spindle microtubules to kinetochore,” and “mitotic spindle assembly checkpoint.” Some genes in batch 2 are also associated with cell division functions, suggesting that altered cell division is consistently associated with *Wolbachia* infection in these samples. Our finding that genes associated with cell division processes are differentially regulated in *Wolbachia*-infected flies is consistent with a previous study, which found that a cell division suppressor protein 14-3-3 zeta was downregulated in *Wolbachia*-infected flies (Christensen *et al.* 2016). Furthermore, the transcriptomic changes to cell division are consistent with a previously documented phenotype associated with *Wolbachia*-increased mitosis in germline stem cells associated with a 4-fold increase in egg production in *Drosophila mauritania* (*D. mauritiana*) (Fast *et al.* 2011). Previous work on 2 strains used in this study, RAL306 and RAL853, also suggest that *Wolbachia* infection is associated with increased reproductive output (Singh 2019).

In contrast to our study, the He *et al*. 2019 study did not identify any GO terms related to cell division functions. The differences in results could be related to the different genotypes used for the different studies. Although our study included multiple genotypes, it did not include the genotype studied previously (He *et al.* 2019). Given that genotype has a large effect on transcription in *D. melanogaster* and that our data indicate transcriptional variation due to infection depends on host genotype, the differences between the 2 studies may result from differences in genotypes employed in the experiments. In addition, *Wolbachia* titer and phenotypes may vary as a consequence of many factors including diet, maternal age, and temperature, to name a few. Though temperature and light–dark cycle appear identical between the 2 studies, sufficient information is not provided to compare the effects of diet and/or maternal age. Thus, these factors may also contribute to the differences between the 2 studies. Future studies should address this variation by including a diversity of genetic backgrounds and environmental conditions.

### Other enriched terms are consistent with previous studies of the effect of *Wolbachia* infection on host transcription

We found a number of other processes enriched in *Wolbachia*-infected flies that can largely be grouped into 2 categories: metabolism and translation. Altered metabolism is frequently associated with host–microbe interactions (Maslowski 2019), and our findings fit this pattern. *Wolbachia* infection is associated with depletion of certain classes of lipids in the mosquito *Aedes albopictus* (Molloy *et al.* 2016), increased host resistance to iron depletion (Brownlie *et al.* 2009), and altered host production of dopamine-dependent arylalkylamine *N*-acetyltransferase (Gruntenko *et al.* 2017). We also found altered *N*-acetylneuraminate catabolism associated with *Wolbachia* infection in our study, along with other metabolic processes such as “negative regulation of insulin secretion,” “glucosamine catabolic process,” “glutathione metabolic process,” and “chitin metabolic process.” Altered metabolism was found in the 2 other studies of *Wolbachia*-infected ovaries of *D. melanogaster*: Christenson *et al*. (2016) found differential levels of proteins related to carbohydrate transfer and metabolism, and He *et al*. (2019) found differential regulation of starch and sucrose metabolism genes.

Apart from altered cell division, we found the processes most affected by *Wolbachia* infection are those related to translation, protein synthesis, and ribosome synthesis. Furthermore, most of these differentially expressed genes are downregulated. These results are consistent with the previous work (Christensen *et al.* 2016), who also found downregulation of protein synthesis proteins in both *Wolbachia*-infected *D. melanogaster* and *D. simulans*. Interestingly, a recent study found that perturbing assembly of translation components through RNAi results in increased *Wolbachia* titer, further supporting a link between decreased translation and *Wolbachia* infection (Grobler *et al.* 2018).

### Genomic location of differentially expressed genes does not support model of increased recombination via global increases in transcription

We found that genes with differential expression in *Wolbachia*-infected flies were randomly distributed across each chromosome arm. We also found that the differentially expressed genes had approximately equal levels of upregulation and downregulation. These findings are in contrast to a model in which recombination increases with *Wolbachia* infection because *Wolbachia* causes global upregulation of genes. We tested this hypothesis because across many eukaryotes, sites of recombination are correlated with sites of increased transcription (Aguilera and Gaillard 2014), and it is proposed that this correlation arises because transcription promotes recombination to resolve genomic instability (Gottipati and Helleday 2009). This correlation exists in *D. melanogaster*, as genes with ovarian transcription above 1 FPKM are concentrated in sites of known recombination (Adrian and Comeron 2012).

If the observed increase in recombination at the X-chromosome locus associated with *Wolbachia* infection (Singh 2019; Mostoufi and Singh 2022) was due to increased transcription in this region, we expect to observe higher numbers of upregulated genes at this region. We instead observe approximately equal numbers of upregulated and downregulated genes at this region. We looked at this region in particular because we know it is associated with increased recombination under *Wolbachia* infection, but it is also possible that *Wolbachia* could increase transcription at other genomic locations we have not yet quantified. However, this trend of randomness extends to the rest of the genome: upregulated genes are distributed evenly across the genome, and we see approximately equal distribution of upregulated and downregulated genes (Fig. 2). The equal distribution of differentially expressed genes across the genome and the similarity in the numbers of up- and downregulated genes does not support the hypothesis that *Wolbachia* infection promotes increased recombination via increased transcription across many loci.

### Differentially expressed genes overlap with previous meiosis studies

It has recently been shown that *Wolbachia* infection also increases the recombination fraction (Singh 2019; Bryant and Newton 2020; Mostoufi and Singh 2022). If *Wolbachia*-associated plastic recombination is driven by changes in gene expression, then genes that are differentially expressed in response to infection may point to the molecular genetic mechanisms underlying increased recombination in *Wolbachia*-infected flies. Thus, we explored these data to identify potential candidate genes that mediate *Wolbachia*-associated plastic recombination. Previous research supports a connection between altered gene expression and altered recombination, as some recombination genes are known to affect recombination in a dosage-sensitive manner (Reynolds *et al.* 2013; Ziolkowski *et al.* 2017). Although genes underlying other the *Wolbachia*-induced cytoplasmic incompatibility phenotype have been identified (Beckmann *et al.* 2017; LePage *et al.* 2017), the genetic basis of *Wolbachia*-associated plastic recombination is unknown.

Since the X-chromosome interval was previously found to exhibit increased recombination in *Wolbachia*-infected flies (Singh 2019; Mostoufi and Singh 2022), we compared our differentially expressed genes to a list of 111 genes significantly associated with variation in recombination at the X-chromosome locus as determined by a GWAS (Hunter *et al.* 2016). We found 10 genes in common between these 2 studies: *Pka-C3*, *px*, *ND-23*, *oat*, *DAAM*, *Tgi*, *dpr6*, *spri*, *Msp3000* (batch 1), and *CG11200* (batch 2). These genes do not have annotated functions in meiosis, suggesting a potential unknown mechanism for modifying recombination rate.

In addition, we found commonalities with a list of 25 genes from a 2002 review paper of experimentally confirmed meiosis genes (McKim *et al.* 2002). Four genes reviewed in this paper were differentially expressed in our study: *c(2)m*, *sub*, *ncd* (batch 1), and *ord* (batch 2). Among the genes involved in early meiosis is *c(2)m*, which is involved in synaptonemal assembly. Since *c(2)m* suppresses crossovers (Manheim and McKim 2003), its significant downregulation in batch 1 may act to release the suppression of crossovers, ultimately resulting in more crossovers during meiosis. We also identified genes known to be involved in late meiosis, *sub* and *ncd*, which both play a role in the formation of the meiotic spindle pole. Though we certainly cannot exclude the possibility that these transcriptional changes are unrelated to plastic recombination given our experimental setup or due to chance, this tractable set of potential candidate genes provides an avenue of further study into the potential mechanisms of *Wolbachia*-induced plastic recombination.

### Conclusions

We investigated how host genotype affects the manner in which ovarian transcription is altered with *Wolbachia* infection in this interesting host–microbe system. We found that *Wolbachia* infection contributes in a limited and variable way to host ovarian transcription. More markedly, we demonstrated for the first time that *Wolbachia*'s effect on ovarian transcription is mediated by the host's genotype. Altered processes include cell cycle, translation, metabolism, cell division, and recombination. These identified transcriptomic changes highlight the importance of host genotype in *Wolbachia*-associated changes in gene expression and will guide future studies of *Wolbachia*'s symbiosis with its *D. melanogaster* host.

## Supplementary Material

jkad047_Supplementary_Data

## Data Availability

Supplemental material available at G3 online.
