# Supplementary material for: Ovarian transcriptional response to Wolbachia infection in D. melanogaster in the context of between-genotype variation in gene expression
Source: G3 (Bethesda). 2023 Mar 1;13(5):jkad047. doi: 10.1093/g3journal/jkad047 (PMC10151400; doi:10.1093/g3journal/jkad047)
Supplement: jkad047_Supplementary_Data [file jkad047_supplementary_data.zip › Supplementary_Figure_1_G3-2022-404021.docx]

1.
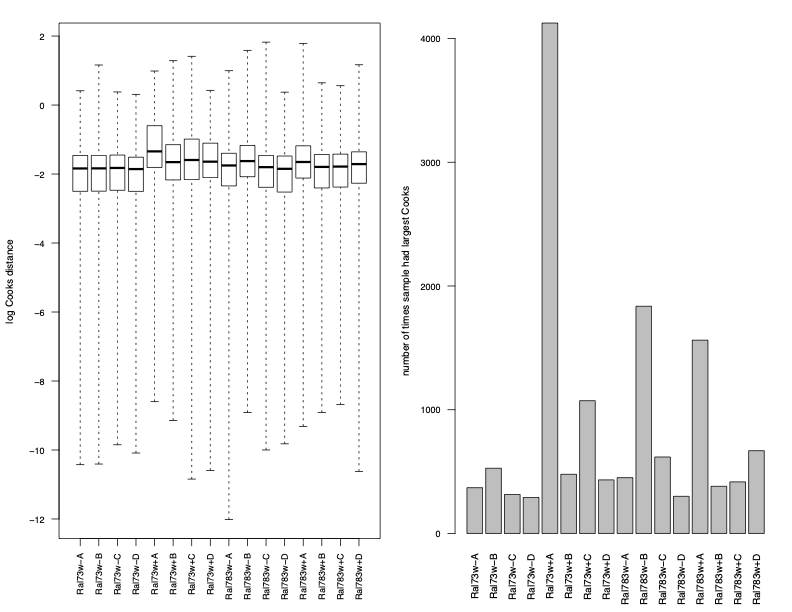
 **b)**

**Supplementary Figure 1:** Outlier investigation through metrics of Cook’s distance. **a)** For every transcript with count data, Cook’s distance was measured for each sample. The distribution of log Cook’s distances for each sample is shown. RAL73w+ has higher average Cooks distance than all other samples. **b)** The number of times each sample had the highest Cook’s distance for a given transcript is plotted. RAL73w+A most often has the highest Cook’s distance.
