# Supplementary material for: Ovarian transcriptional response to Wolbachia infection in D. melanogaster in the context of between-genotype variation in gene expression
Source: G3 (Bethesda). 2023 Mar 1;13(5):jkad047. doi: 10.1093/g3journal/jkad047 (PMC10151400; doi:10.1093/g3journal/jkad047)
Supplement: jkad047_Supplementary_Data [file jkad047_supplementary_data.zip › Supplementary_Table_1_G3-2022-404021.docx]

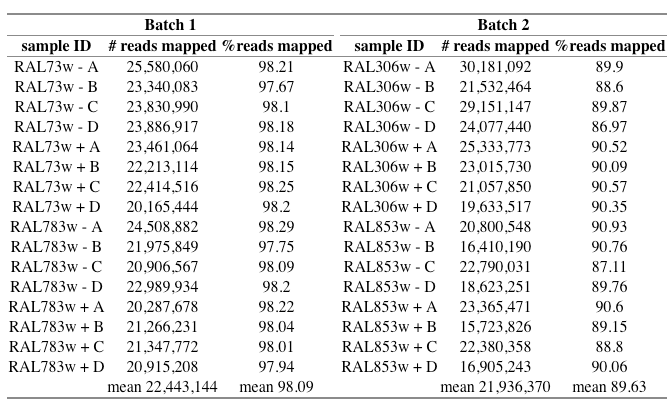
**Supplementary Table 1:**Sequencing Statistics. Shown are the sample ID for batch 1 and batch 2, the total number of reads that mapped uniquely to the genome for each sample, and the percent of total reads that mapped uniquely to the genome. The mean number and percentage across samples in each batch are also shown.
