# Supplementary material for: Ovarian transcriptional response to Wolbachia infection in D. melanogaster in the context of between-genotype variation in gene expression
Source: G3 (Bethesda). 2023 Mar 1;13(5):jkad047. doi: 10.1093/g3journal/jkad047 (PMC10151400; doi:10.1093/g3journal/jkad047)
Supplement: jkad047_Supplementary_Data [file jkad047_supplementary_data.zip › Supplementary_Table_2_G3-2022-404021.docx]

**Supplementary Table 2:**Pairwise relatedness for the four lines used in the current study. Data are from <http://dgrp2.gnets.ncsu.edu/data.html>

| Line | 73 | 783 | 306 | 853 |
| --- | --- | --- | --- | --- |
| 73 |  |  |  |  |
| 783 | 0.047 |  |  |  |
| 306 | -0.0066 | -0.0030 |  |  |
| 853 | 0.0040 | -0.0026 | -0.011 |  |
